# Suppressor of fused (Sufu) promotes epithelial-mesenchymal transition (EMT) in cervical squamous cell carcinoma

**DOI:** 10.18632/oncotarget.23176

**Published:** 2017-12-11

**Authors:** Ziyu Zhang, Yang Zou, Meirong Liang, Yuanting Chen, Yong Luo, Bicheng Yang, Faying Liu, Yunna Qin, Deming He, Feng Wang, Ouping Huang

**Affiliations:** ^1^ Key Laboratory of Women's Reproductive Health of Jiangxi, Jiangxi Provincial Maternal and Child Health Hospital, Nanchang, Jiangxi 330006, P.R. China; ^2^ Graduate School of Nanchang University, Nanchang, Jiangxi 330031, P.R. China; ^3^ Department of Pathology, Jiangxi Provincial Maternal and Child Health Hospital, Nanchang, Jiangxi 330006, P.R. China

**Keywords:** cervical carcinoma, Sufu, 14-3-3ζ, EMT, FoxM1

## Abstract

Suppressor of fused is essential for the maximal activation of Sonic Hedgehog signaling in development and tumorigenesis. However, the role of Sufu in cervical carcinoma remains unknown. Here, we report new findings of Sufu in regulating the epithelial-to-mesenchymal transition through the FoxM1 transcriptional modulation by 14-3-3ζ protein in cervical carcinoma. Sufu is overexpressed in cervical squamous cell carcinoma and its level in clinical tumor tissues is positively correlated with 14-3-3ζ. Functionanlly, siSufu remarkably prevents the cancer cell migration and invasion. We further demonstrate that the transcriptional activity of Sufu is increased by FoxM1, of which stability is promoted by 14-3-3ζ. Knockdown FoxM1 decreases the invasion of SiHa cells and reconstitution of Sufu rescues the invasion of these cells.Finally, overexpression of Sufu is significantly associated with differentiation grade, FIGO stage, Depth of stromal invasion and vascular cancer embolus. Our findings highlight a novel role for Sufu in cervical carcinogenesis.

## INTRODUCTION

Hedgehog (Hh) signaling pathway plays a critical role in the development, especially in the developing of neural tube and limb bud [1–5]. Besides, abnormal Hh signaling activity was found in tumorigenesis, for example, medulloblastoma (MB)and basal cell carcinoma (BCC) [6]. Suppressor of fused (Sufu) has been known as the Hh signaling pathway inhibitor, which is considered as a tumor suppressor in early studies [7–9]. However, a surprising observation was made that Sufu was required for maximal activation of Hh signaling [10, 11]. Recently, more and more evidence has shown that aberrant activation of Hh signaling pathway is involved in the formation of respiratory system malignance, gastrointestinal tumor and reproductive system cancers [12–14]. In gynecologic carcinoma, the pathogenesis of cervical cancer is effected by the alterations of Hh pathway regulators [15–17], but the molecular mechanism remains unclear.

14-3-3 protein family is one of the widely expressed conserved regulatory proteins in eukaryotic cells, which consists of seven family members (β, ε, γ, ζ, σ, τ, and δ). One of the most striking features of these proteins is the ability to interact with many functional signaling proteins, including kinases, phosphatases and transmembrane receptor proteins [18–20]. In some cases, 14-3-3 proteins bind to other signal molecules via phosphorylation-dependent manner by its canonical sequence motifs [RSxpSxP and RxxxpSxP] and non-canonical recognition sequences [21]. 14-3-3 proteins promoted tumorigenesis by affecting cell proliferation, apoptosis and epithelial-to-mesenchymal transition(EMT) because of mediating the biological function of its binding partners [22].

Here, we present evidence that 14-3-3ζ is associated with FoxM1, a forkhead transcription factor and is required for FoxM1 stability. We identify that FoxM1 activates Sufu expression by binding to Sufu promoter. Functionally, Sufu knockdown prevents cervical cancer cell EMT *in vitro*. These data suggest that 14-3-3ζ induces EMT in Cervical squamous cell carcinoma (CSCC) through FoxM1-Sufu/Hh cascade.

## RESULTS

### Knockdown Sufu inhibits invasion and migration of cervical cancer cells *in vitro*

**Figure 1:**
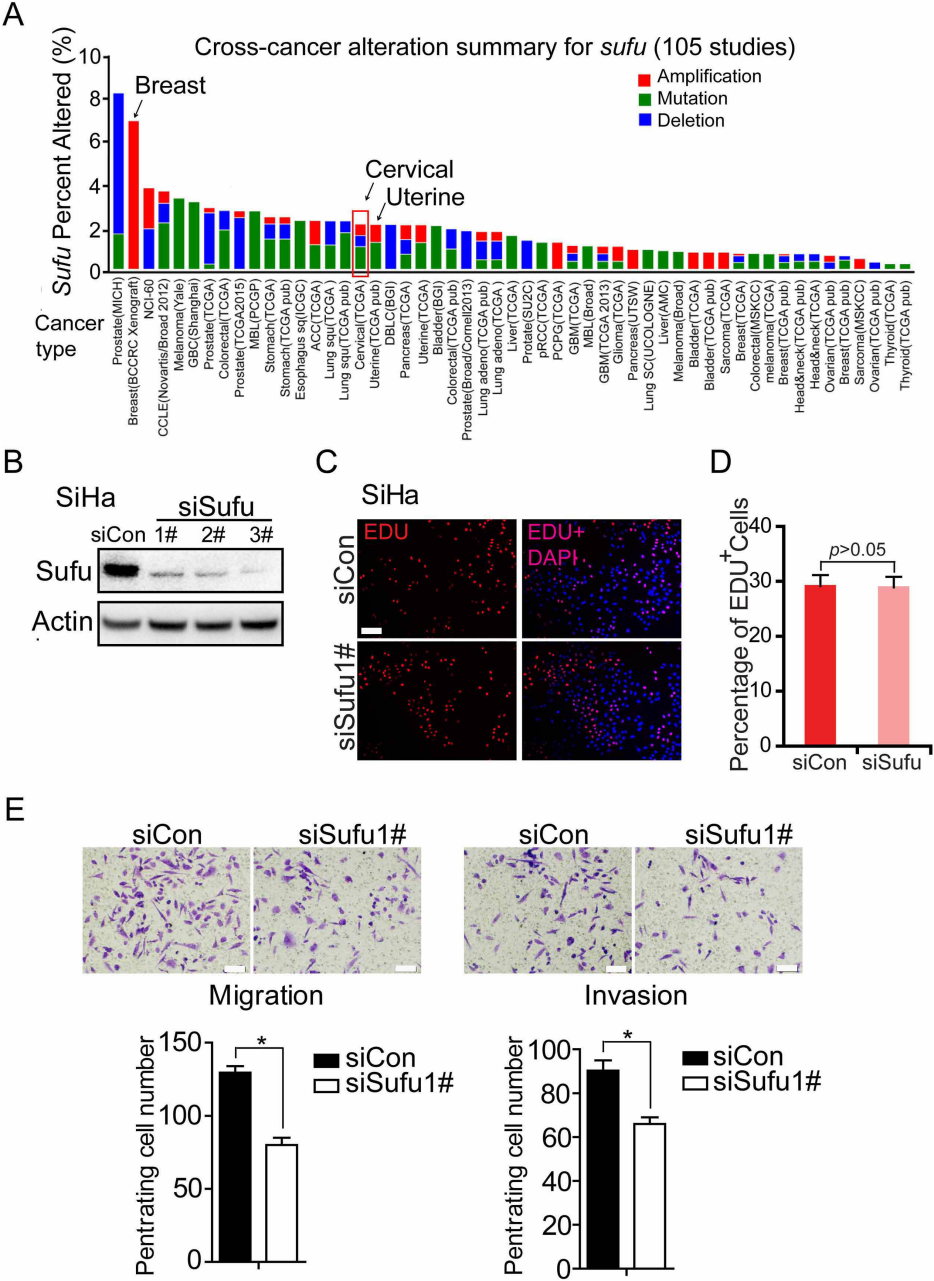
Knockdown Sufu inhibits invasion and migration of cervical cancer cells *in vitro* **(A)** Multidimensional cancer genomics data analysis showing cross-cancer Sufu gene alteration. The histogram showed the frequencies of Sufu gene mutation, deletion, and amplification across cancers. Data were extracted from TCGA database and analyzed using cBioPortal online analyzing tools. **(B)** Western analysis of SiHa showing the knockdown effect of siSufu. **(C)** EdU incorporation assays of SiHa cells transiently transfected with control siRNA or siSufu. **(D)** The percentage of EdU positive cells was blindly calculated with counting nine nonoverlaping fields. Values are means±s.d. **(E)** SiHa cells were transfected with control siRNA or siSufu. After 24 hours of transfection, cells were starved for 24h before cell migration and invasion assays were performed with or without matrigel transwell filters. The invaded or migrated cells were stained and counted. Each bar indicates mean±s.d. of a representative experiment performed in triplicate. P-values were determined by Student’s t-test. ^*^ p<0.05.

To determine whether Sufu plays a positive role in carcinogenesis, we analyzed Sufu gene alteration using data from The Cancer Genomic Atlas (TCGA) database and the cBioPortal online tool (the cBioPortal for Cancer Genomics) [23, 24]. Interestingly, among 53 examined cancer types or subtypes(from a total of 105 studies), the cervical cancer has Sufu gene amplification (Figure 1A). To investigate the function of Sufu during cervical cancer progression, we used cervical squamous cancer line to clarify the role of Sufu in cell proliferation, invasion and migration. Firstly, we compared the effects of Sufu knockdown and control siRNA on the proliferation of SiHa cells using EdU incorporation experiment. Western blot analysis indicated that Sufu siRNA successfully reduced Sufu protein level in SiHa cell (Figure 1B). However, knockdown of Sufu protein did not alter SiHa cell proliferation (Figure 1C, 1D). We then performed transwell migration assay and matrigel invasion assay in SiHa and HCC94 cell. Interestingly, as shown in Figure 1E and Supplementary Figure 1, Sufu knockdown not only significantly inhibited the cell migration, but also reduced the cell invasion. These results suggest that knockdown of Sufu by siRNA attenuated cervical cancer cell migration and invasion, but not proliferation.

### Sufu promoters EMT in SiHa cell but not in HeLa cell

**Figure 2:**
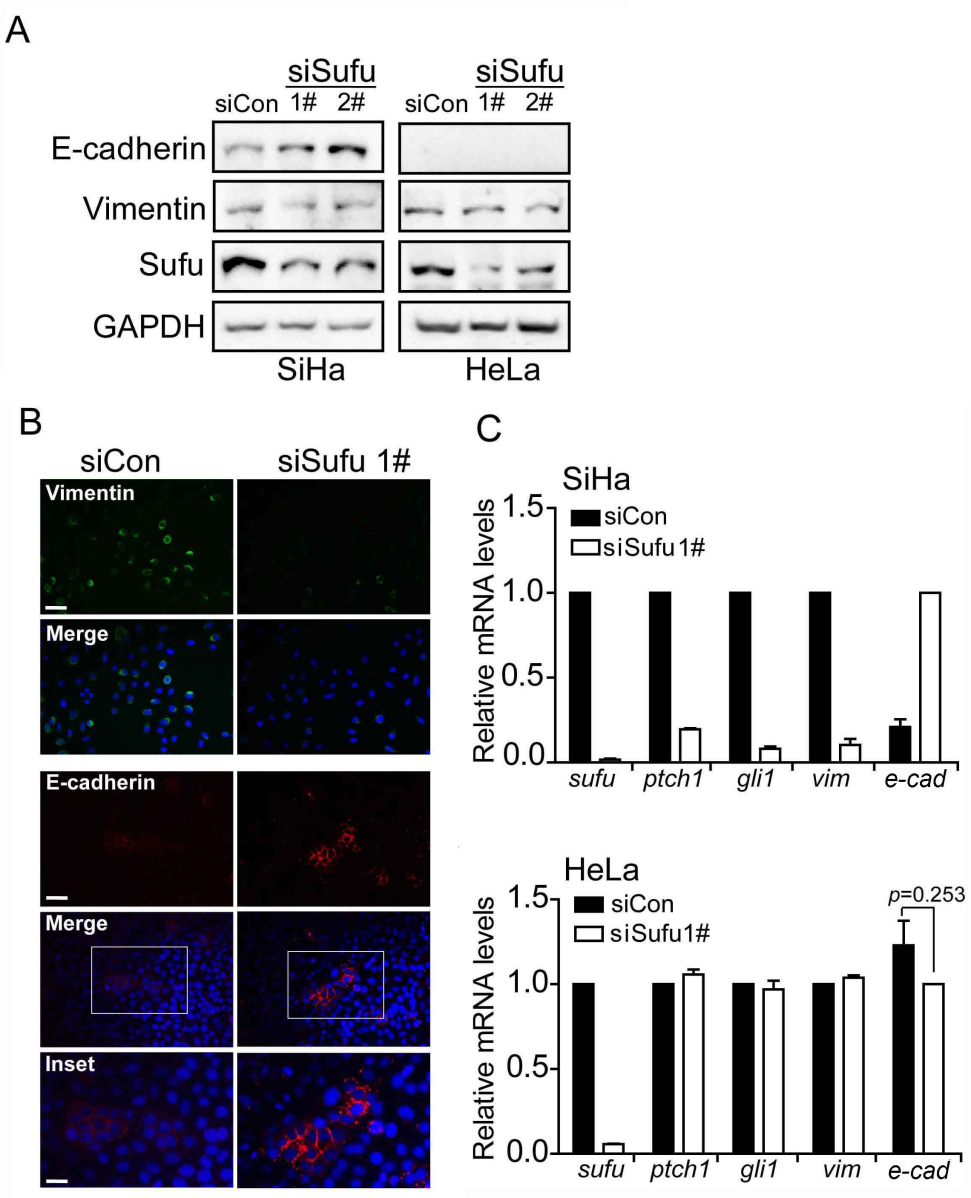
Sufu regulates EMT proteins in SiHa cells but not in HeLa cells **(A)** Sufu depletion led to an elevation or a reduction in E-cadherin or Vimentin protein level in SiHa cells but not in HeLa cells, as measured by western blotting. GAPDH was used as the loading control. **(B)** SiHa cells were transfected with control siRNA or siSufu. Immunofluorescence staining of epithelial (E-cadherin, Red) and mesenchymal (Vimentin, Green) markers was visualized by microscopy. DAPI staining was included to visualize the cell nucleus (Blue). Scale bar=100 μm. **(C)** Sufu was silenced by siRNA in SiHa cells (top) and HeLa cells (bottom), respectively. The EMT markers and Hedgehog pathway target genes were measured by qPCR. Each bar indicates mean±s.d. of three independent experiments.

Based on the above studies, Sufu is considered to be a master regulator of EMT. To this end, we examined the expression levels of EMT markers in SiHa and HeLa. As expected, in SiHa cell, western blot analysis showed that Sufu knockdown indeed up-regulated the E-cadherin protein level and down-regulated the Vimentin protein level, respectively (Figure 2A). Nevertheless, depletion of Sufu did not change the protein levels of the EMT markers in HeLa cell, which is a cervical adenocarcinoma cell line. Meanwhile, once we transfected Sufu siRNA into SiHa cells, the mRNA expression of Hedgehog signaling downstream target genes Ptch1 and Gli1 was obviously decreased as well as Vimentin mRNA expression (Figure 2C). On the contrary, mRNA level of E-cadherin was increased by siSufu. However, neither the mRNA levels of EMT markers nor the protein levels of EMT markers were altered by silencing Sufu in HeLa cells (Figure 2A, 2C). Besides, our immunofluorescence (IF) experiment using anti-Vimentin and anti-E-cadherin antibody to detect endogenous Vimentin and E-cadherin showed that both of their protein levels were changed once Sufu knockdown in SiHa (Figure 2B).

### 14-3-3ζ regulates the EMT markers through mediating FoxM1-Sufu/Hedgehog cascade

**Figure 3:**
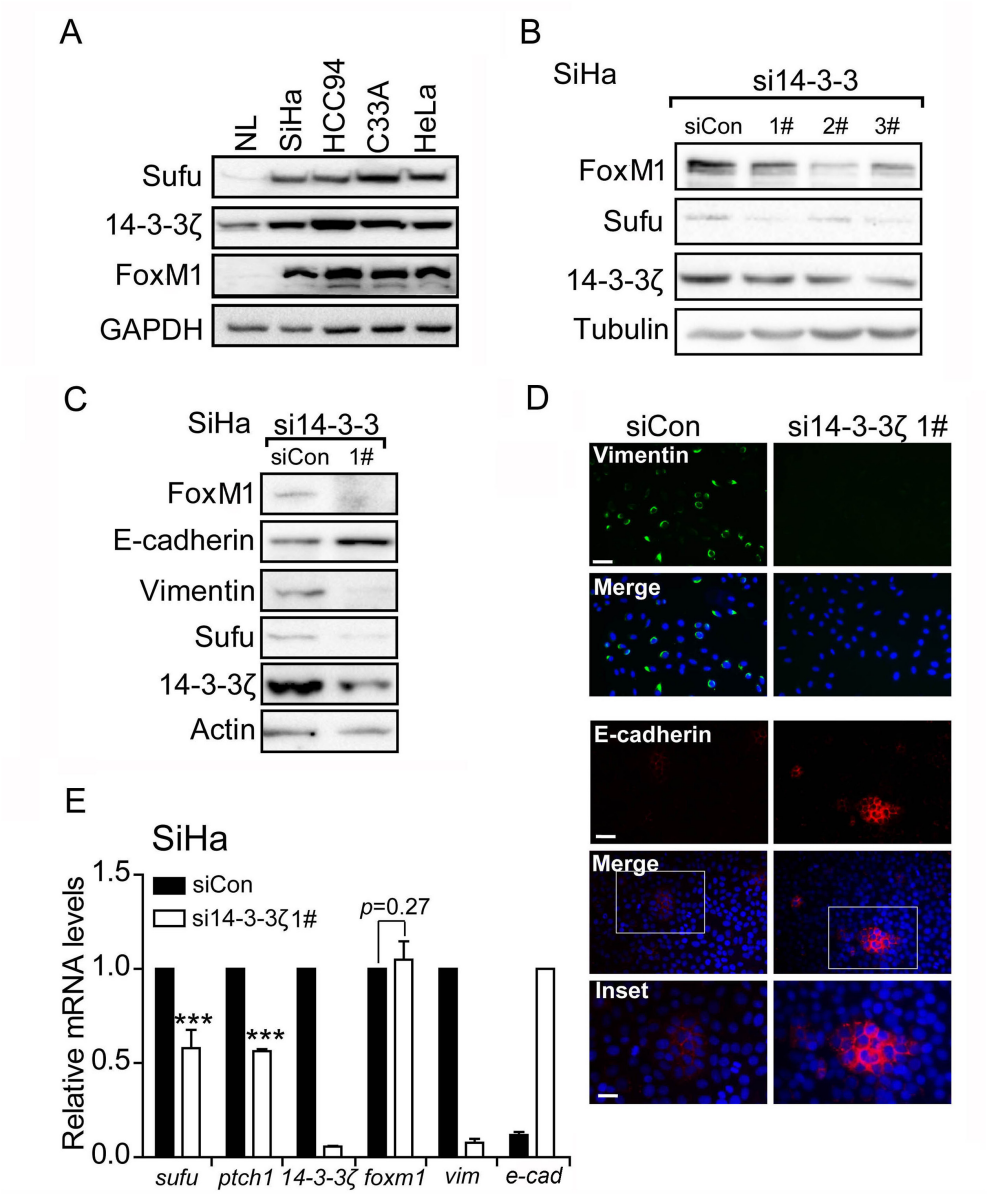
14-3-3ζ regulates the EMT markers through mediating FoxM1-Sufu/Hedgehog cascade **(A)** Western blot analysis of Sufu, 14-3-3ζ and FoxM1 protein levels in total lysates of human normal tissue (NL) and human cervical cancer cells SiHa, HCC94, C33A and HeLa. GAPDH was used as the loading control. **(B)** SiHa cells were transfected with control siRNA or si14-3-3ζ. Sufu and FoxM1 protein levels were measured by western blot. **(C)** Western blot analysis showing effects of si14-3-3ζ on protein levels of EMT markers. **(D)** Immunofluorescence staining showing effects of si14-3-3ζ on expression levels of Vimentin and E-cadherin. Scale bar=100 μm. **(E)** 14-3-3ζ was silenced by siRNA in SiHa cells. The EMT markers and Hedgehog pathway target genes were measured by qPCR. Each bar indicates mean±s.d. of three independent experiments. ^***^ p<0.001.

Western blot analysis showed that Sufu was highly expressed in cervical cancer cell lines (SiHa, HCC94, C33A and HeLa), while in normal tissue (NL), Sufu protein was expressed at low level (Figure 3A). To investigate the mechanism of high level of Sufu in cervical cancer cell lines, we focus on the 14-3-3ζ protein, which is a binding partner through a phosphorylation-dependent manner. Using the online search tool 14-3-3-Pred (http://www.compbio.dundee.ac.uk/1433pred) [25], we identified a sequence motif in Sufu (RAPS^342^RKDS^346^) that closely matches to the 14-3-3 binding consensus RX_1-2_SX_2-3_S [21].Moreover, the S342 and S346 sites were previously reported and required for Sufu stabilization and nuclear accumulation [10, 26]. Accordingly, we examined the 14-3-3ζ protein level in NL and cervical cancer cell lines. Compared to NL, 14-3-3ζ protein level was overexpressed in several cervical cancer cell lines (Figure 3A). Therefore, we speculated that 14-3-3ζ induced Sufu expression level. Indeed, 14-3-3ζ knockdown abrogated Sufu protein (Figure 3B). However, we failed to detect any binding signal by Co-IP experiment (Supplementary Figure 2). Interestingly, si14-3-3ζ down-regulated both the Sufu and Hedgehog target genes mRNA expression levels (Figure 3E), indicating that 14-3-3ζ regulated Sufu expression on the transcriptional level. Besides, expression levels of EMT markers were also affected by si14-3-3ζ in SiHa cell (Figure 3C, 3E, 3D) and HCC94 cell (Supplementary Figure 3).

14-3-3ζ is a molecular chaperone but not a transcriptional factor, so we speculate that 14-3-3ζ induces Sufu mRNA level through an intermediator. FoxM1, a transcriptional factor and 14-3-3ζ downstream target [27], is associated with cervical cancer progression and pathogenesis [28]. To determine whether FoxM1 is regulated by 14-3-3ζ in cervical cancer cell line, knockdown 14-3-3ζ reduced FoxM1 protein level (Figure 3B, Supplementary Figure 4) rather than mRNA level (Figure 3E). Additionally, data performed by ENCODE project derived from a large collection of ChIP-seq experiments revealed that a putative FoxM1 recognition sequence (TAAACA/TGTTTA) within Sufu promoter [29], indicating that Sufu is a potential FoxM1 direct downstream target gene (http://genome.ucsc.edu/cgi-bin/hgGateway). Therefore, we hypothesizes that 14-3-3ζ regulates Sufu/Hedgehog pathway through mediating FoxM1.

### Inhibition of 14-3-3ζ decreases migration and invasion of cervical cancer cell

**Figure 4:**
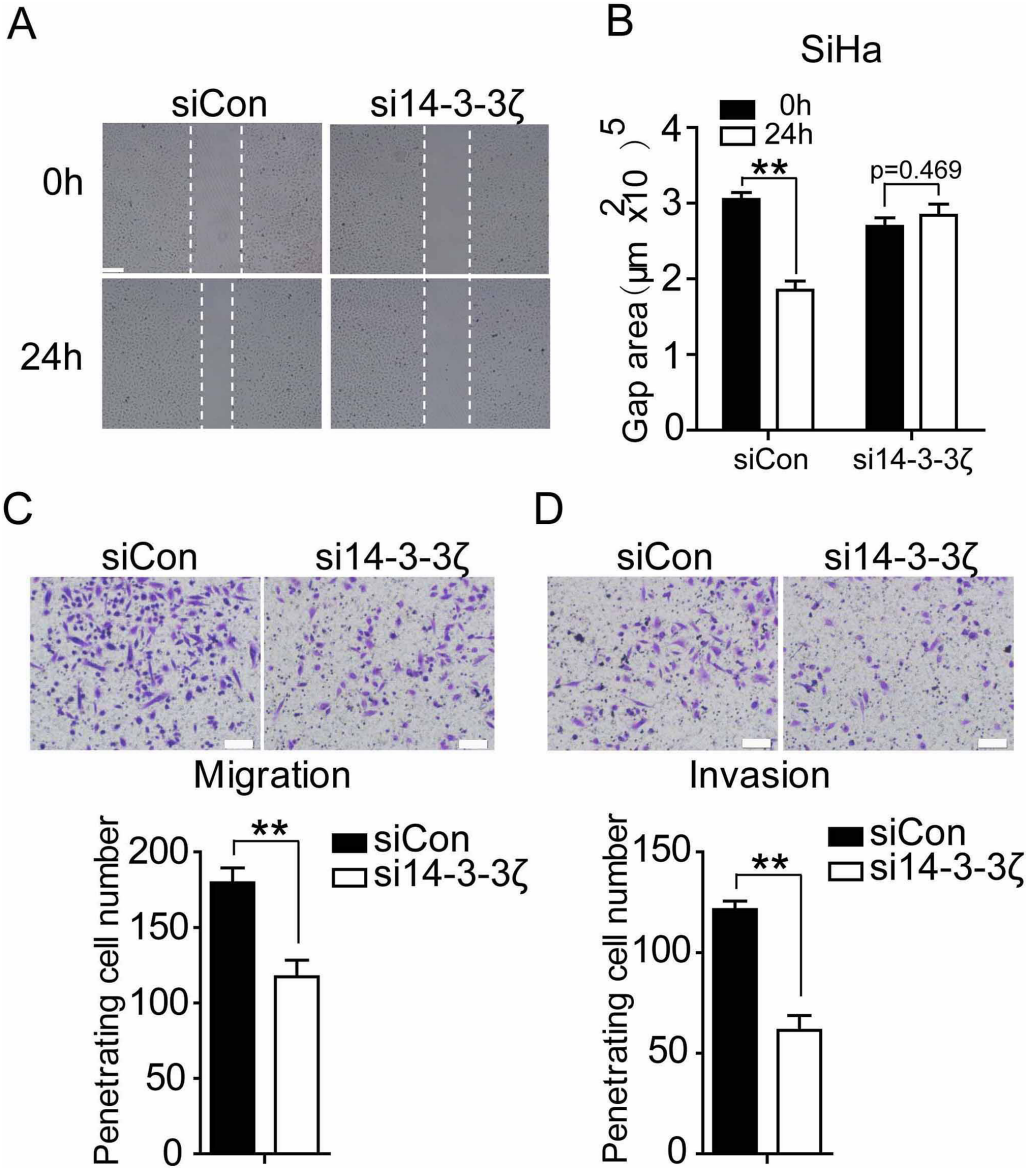
Inhibition of 14-3-3ζ decreases migration and invasion of SiHa cell **(A)** Wound-healing assay showing that si14-3-3ζ inhibits SiHa cells migration within 24 hours in serum-free medium. Scale bar=200 μm. Quantification of (A) was done in and is shown in **(B)** with counting nine nonoverlaping fields. **(C** and **D)** SiHa cells were transfected with control siRNA or si14-3-3ζ. After 24 hours of transfection, cells were starved for 24h before cell migration and invasion assays were performed with or without matrigel transwell filters. The invaded or migrated cells were stained and counted. Each bar indicates mean±s.d. of a representative experiment performed in triplicate. P-values were determined by Student’s t-test. ^**^ p<0.01.

As the above investigation discovered that 14-3-3ζ elevated EMT in cervical cancer cell, we next examined its effect on the cell migration of cervical cancer cell by wound healing assay and transwell migration assay *in vitro*. Depletion of 14-3-3ζ expression in SiHa and HCC94 caused significant inhibition on motility as compared with empty vector control (Figure 4A, 4B, 4C, Supplementary Figure 5A). We examined the effect of 14-3-3ζ on the invasion of cervical cancer cell using a matrigel invasion assay. SiHa and HCC94 cell with decreased 14-3-3ζ level showed significant inhibition of penetrating cell number (Figure 4D, Supplementary Figure 5B). These data support a role for 14-3-3ζ in promoting cervical cancer cell migration and invasion.

### 14-3-3ζ is required for FoxM1 stabilization

**Figure 5:**
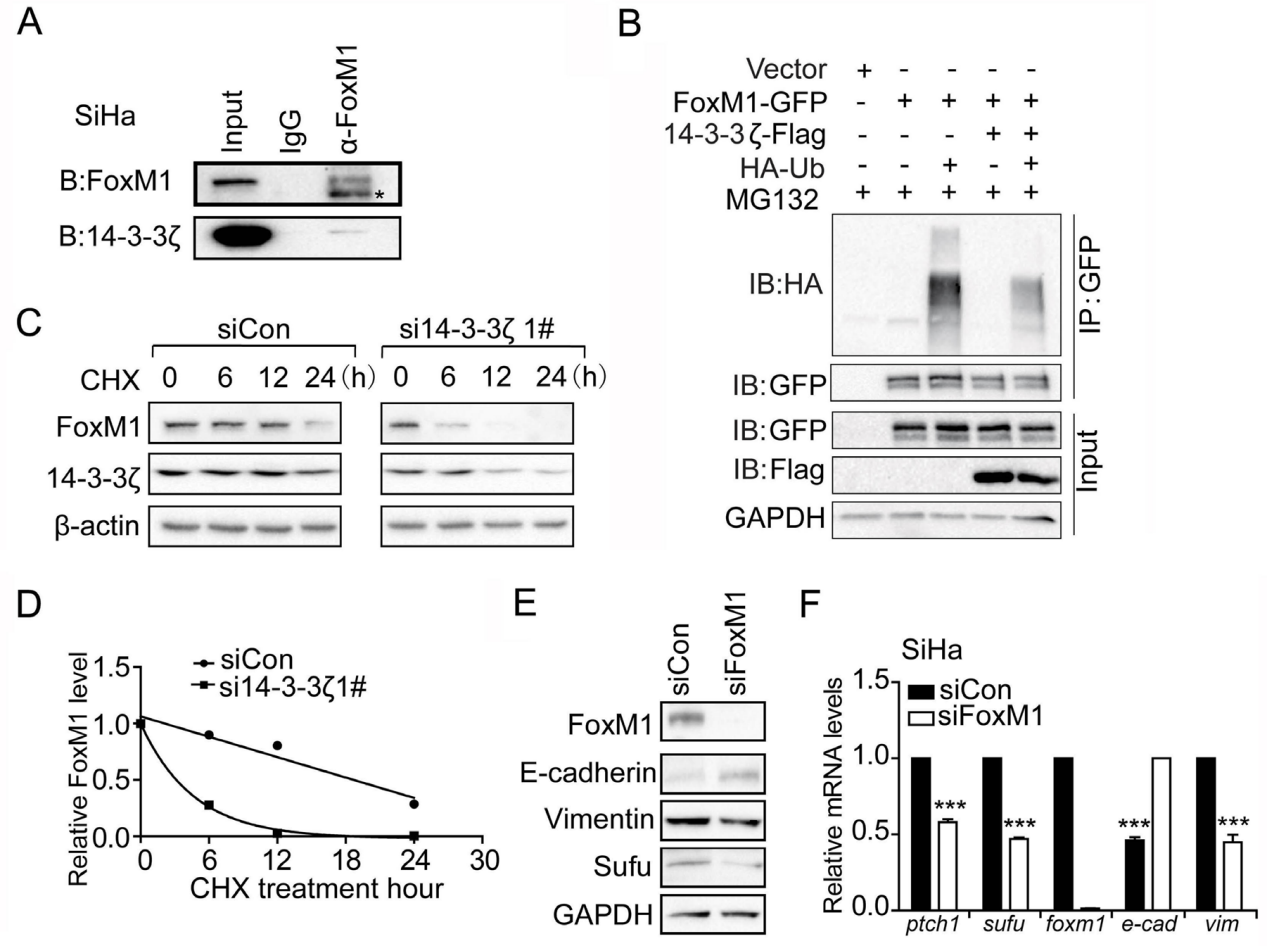
14-3-3ζ regulates Sufu mRNA level through promoting FoxM1 stability **(A)** Co-IP experiment showing endogenous proteins interaction between 14-3-3ζ and FoxM1 in SiHa cells. **(B)** Detection of polyubiquitylated species of FoxM1 upon co-transfection of HEK293T cells with Ha-tagged ubiquitin (Ub) either in the presence of 14-3-3ζ or control vector. **(C)** Western analysis of FoxM1 in SiHa cells following cycloheximide (CHX, 20um) treatment in the presence of siControl or si14-3-3ζ. **(D)** Time course of FoxM1 turnover experiments as described in (C). The intensity of FoxM1 bands relative to that of tubulin from each time point was quantified by ImageJ software package and plotted against the incubation time. **(E)** Western blot analysis showing effects of siFoxM1 on protein levels of Sufu and EMT markers. GAPDH was used as the loading control. **(F)** FoxM1 was silenced by siRNA in SiHa cells. The EMT markers and Hedgehog pathway target genes were measured by qPCR. Each bar indicates mean±s.d. of three independent experiments. ^***^ p<0.001.

Since FoxM1 mRNA level was not altered by 14-3-3ζ knockdown (Figure 3E), but its protein level was decreased (Figure 3C). Additionally, 14-3-3ζ enhances protein levels through promoting their stabilization [30]. This interaction between 14-3-3 protein family and their target proteins is mediated by a conserved amphipathic groove, like a dimeric structure, which is thought to be consisted of two 14-3-3 proteins [21]. Again, we found several phosphorylation sites in FoxM1 predicted by 14-3-3-Pred software. Therefore, we speculated that 14-3-3ζ induced FoxM1 protein expression level by promoting FoxM1 stabilization. To that end, we measured the rate of FoxM1 decay after blocking protein synthesis with cycloheximide (CHX). Under the condition of 14-3-3ζ–depleted, FoxM1 protein level rapidly reduced to half of its original amount at 6 h, but no significant change was observed at this time point with control siRNA treatment (Figure 5C, 5D). Furthermore, our co-IP experiment showed that endogenous 14-3-3ζ was brought down by FoxM1 (Figure 5A). Remarkably, polyubiquitylation of exogenous FoxM1 was impeded by the presence of exogenous 14-3-3ζ (Figure 5B). These results suggested that 14-3-3ζ is required for FoxM1 stabilization by inhibiting ubiquitin-mediated proteasome degradation.

### Sufu is a direct downstream target gene of FoxM1

**Figure 6:**
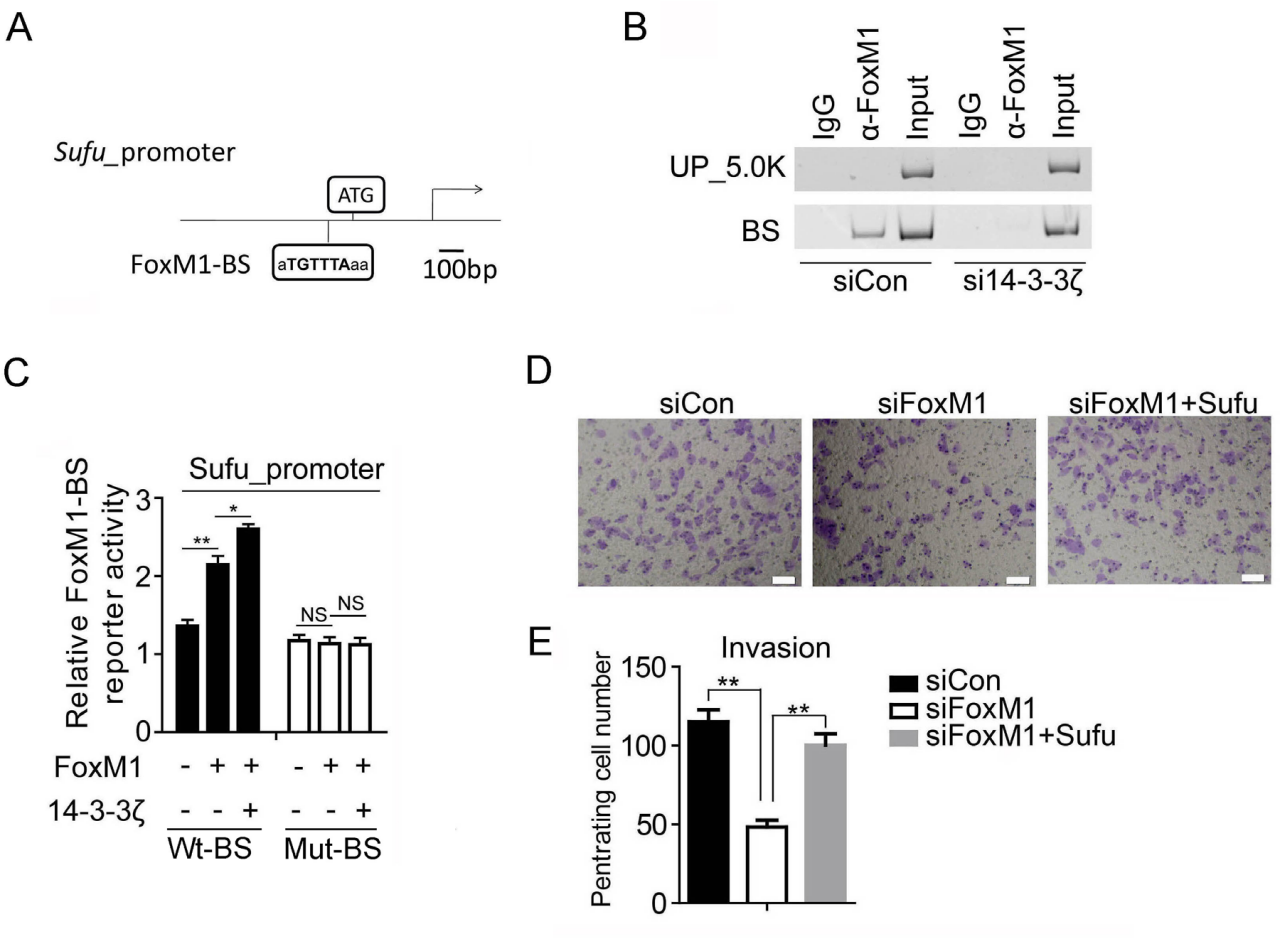
Sufu is a direct downstream target gene of FoxM1 **(A)** Schematic drawing showing the Sufu promoter region including two FoxM1-binding sites (BS). **(B)** PCR analysis of FoxM1 ChIP products in the Sufu promoter regions in SiHa cells. **(C)** Sufu luciferase constructs, as indicated, were transfected into 293T cells together with indicated plasmid for 48h and subjected to a luciferase reporter assay. The results were normalized to the Renilla luciferase activity and are expressed as the fold change in relative luciferase activity compared with the control. Error bars represent the mean and S.D. of three independent experiments. **(D)** HCC94 cells were transfected with control siRNA or siFoxM1 or siFoxM1 and Sufu plasmid. After 24 hours of transfection, cells were starved for 24h before cell invasion assays were performed with or without matrigel transwell filters. The invaded or migrated cells were stained and counted in **(E)**. Each bar indicates mean±s.d. of a representative experiment performed in triplicate. P-values were determined by Student’s t-test. ^*^p < 0.05, ^**^p < 0.01.

To test whether Sufu is a downstream target gene of FoxM1, we used FoxM1 siRNA to analyses the change of Sufu protein level. Consequently, Sufu protein level dramatically decreased after transfected with FoxM1 siRNA (Figure 5E). Simultaneously, the mRNA expression levels of Sufu, Hedgehog target genes and EMT markers were also significantly altered (Figure 5F). To understand how FoxM1 regulates Sufu expression, we used anti-FoxM1 antibody or normal mouse IgG to immunoprecipitation chromatin in SiHa cell. Compared to IgG immunoprecipitates and upstream 5kb region of Sufu promoter, a strong amplification band was obtained from anti-FoxM1antibody immunoprecipitates in the FoxM1 binding region (Figure 6A, 6B). Importantly, there was less binding of FoxM1 on Sufu promoter with 14-3-3ζ depletion (Figure 6A, 6B). Expressing exogenous14-3-3 enhanced the activation of FoxM1-mediated transcription, but there was no effect on the mutant FoxM1 binding site (Figure 6C). In addition, knockdown of FOXM1 expression decreased the invasion of SiHa cells relative to that of control cells, and reconstitution of Sufu restored the invasion of these cells (Figure 6D, 6E). Taken together, our data indicated that FoxM1 regulated Sufu by binding to Sufu promoter directly and participating in Sufu transcription activity.

### Sufu and 14-3-3ζ are positively correlated in human tumor tissues

**Figure 7:**
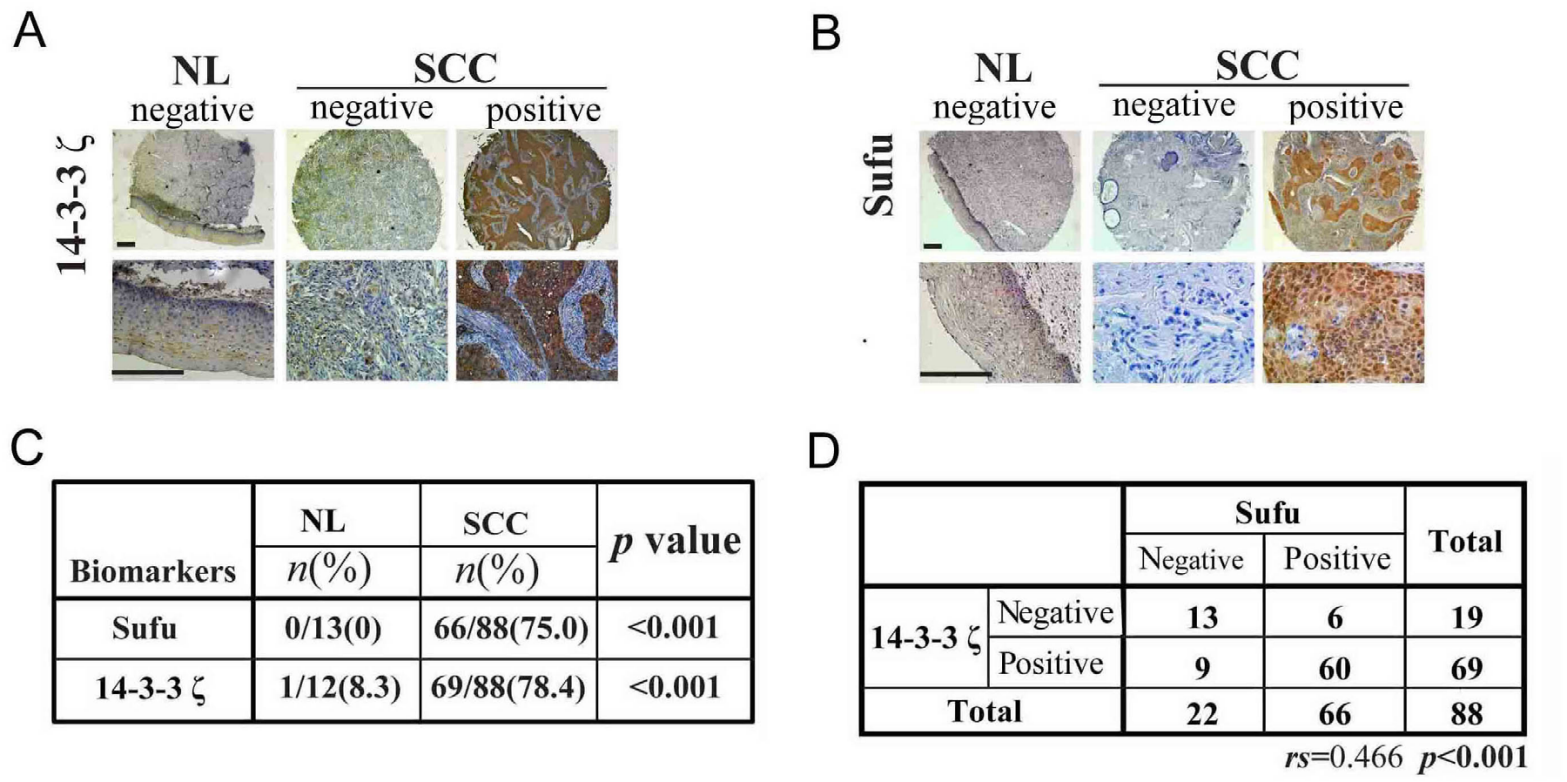
Sufu and 14-3-3ζ are positively correlated in human tumor tissues **(A)** A representative image showing the expression of 14-3-3ζ in NL and CSCC tissues. Scale bar=400μm. **(B)** A representative image showing the expression of Sufu in NL and CSCC tissues. Scale bar=400μm. **(C)** Statistic analysis of immunohistochemistry (IHC) staining of Sufu and 14-3-3ζ from human CSCCtissue microarray. **(D)** Statistic analysis for Sufu and 14-3-3ζ correlation from the IHC staining results in human CSCCtissue microarray.

To investigate the significance of the Sufu regulation by 14-3-3ζ and FoxM1, we evaluated the levels of Sufu, 14-3-3ζ and FoxM1 in human cervical tissue microarray by immunohistochemistry (IHC). Representative IHC staining of Sufu, 14-3-3ζ and FoxM1 in CSCC tissue microarray is shown in Figure 7A, 7B and Supplementary Figure 6A. Positive expression of these proteins was found in most CSCC cases: 66 out of the 88(75.0%) for Sufu, 69 out of 88(78.4%) for 14-3-3ζ (Figure 7C) and 60 out of the 88(68.2%) for FoxM1(Supplementary Figure 7B), and there was a strong correlation between the expression levels of Sufu and 14-3-3ζ (Figure 7D), 14-3-3ζ and FoxM1(Supplementary Figure 6C), Sufu and FoxM1(Supplementary Figure 6D). By contrast, positive expression of both proteins was hardly found in NL cases (Figure 7C, Supplementary Figure 6B). Meanwhile, we also evaluated the expression level of Gli2, a terminal transcription factor in Hedgehog signaling, was overexpressed in CSCC tissue (Supplementary Figure 7). This means that Hh signaling is indeed over activated. Besides, the overexpression of Sufu was correlated with differentiation grade (p=0.008), FIGO stage (p=0.004), depth of stromal invasion (p=0.041) and vascular cancer embolus (p=0.046), no significant correlation was observed between Sufu and other clinicopathological factors, including age (p=0.198), lymph node metastasis (p=0.358) (Table 1).

**Table 1:** Relationship between Sufu expression and clinic pathological characteristics in CSCC

Variables	No. of patients (n=88)	Sufu positive (%)	*p* value
Age			0.198
<40	30	25 (83.3%)	
≥40	58	41 (70.7%)	
Differentiation grade			0.008
1	16	8(50.0%)	
2	69	55(79.7%)	
3	3	3(100.0%)	
FIGO stage			0.004
I	77	59 (76.7%)	
II	11	7 (69.6%)	
Depth of stromal invasion			0.041
<1/2	37	22 (59.5%)	
≥1/2	51	44 (86.2%)	
Vascular cancer embolus			0.046
No	52	35 (67.3%)	
Yes	36	31 (86.1%)	
Lymph node metastasis			0.358
No	77	55 (71.4%)	
Yes	11	11 (100.0%)	

## DISCUSSION

Sufu is involved in the Hh signaling pathway maximal activation [10]. Furthermore, the data from the TCGA database showed that alters of Sufu in cervical cancer have a certain proportion of the amplification (Figure 1A). Accordingly, we speculate that the novel function of Sufu also plays a positive role in the development of cervical cancer. Firstly, we transiently transfected Sufu small interfering RNA into cervical cancer cells (Figure1B) to perform EdU insertion experiments, however, we found no effect of silencing Sufu on cell proliferation (Figure 1C, 1D). Then, we carried out transwell experiments to study the effect of Sufu on migration and invasion in SiHa cells, and Sufu knockdown slowed the motility and invasiveness of cervical squamous cell carcinoma SiHa cells (Figure 1E, 1F). CSCC originates from cervical epithelial cell, which is gradually from epithelial carcinoma *in situ* invading into stromal, and then metastasize to other parts of the body, the characteristics of which is very similar with EMT [31]. We identified that knockdown of Sufu in SiHa cells downregulated the Vimentin (an interstitial cell marker) and upregulated the E-cadherin (an epithelial cell marker) expression (Figure 2). Additionally, previous studies have reported that Hh pathway is frequently overactivated in cervical cancer [16, 32] and we also discovered that Sufu was overexpressed in cervical cancer cell lines (Figure 3A).

However, there are different between squamous cell carcinoma and adenocarcinoma in EMT regulated by Sufu. We also found that Sufu knockdown reduced Hh downstream target genes Gli and Ptch1 (Figure 2C) in cervical squamous cell lines (SiHa and HCC94) rather than in adenocarcinoma cell line (HeLa) (Figure 3A, 3D). Wound healing assay revealed that Sufu siRNA did not change the migration of HeLa cell (Supplementary Figure 8). These results suggest that depletion of Sufu decreases Hh signaling pathway activity and only inhibits EMT in cervical squamous cell lines. We consider that the Hh signaling is destructed between Sufu and Gli in HeLa. Maybe mutations are produced in Sufu or Gli, resulting in the interaction between them does not exist. Subsequent work will be performed to investigate the exact mechanism in order to understand the importance of Sufu in distinguishing cervical squamous cell carcinoma and cervical adenocarcinoma cell carcinoma.

Here, we consider the Sufu as a downstream target of 14-3-3ζ is based on three reasons: 1) 14-3-3ζ is highly expressed in many tumor types and binds to target proteins by a phosphorylation manner [21]; 2) Sufu contains a conserved 14-3-3 binding motif, of which S342 and S346 sites have also been reported to be phosphorylated [26]; 3) 14-3-3-Pred software predicts the two sites are the candidates of 14-3-3 binding sites. Through the above three points, we suppose that Sufu is a potential target for the 14-3-3. But unfortunately, we fail to find any binding signal between Sufu and 14-3-3ζ in co-IP experiment (Supplementary Figure 1). We consider this is likely because the wild type Sufu is hardly interacted with 14-3-3ζ, so we make use of sustained phosphorylated Sufu mutations (S342D, S346D, S342/6D) to carry out Co-IP experiment again, but we are unable to find any combination of signal (data not shown). However, it is very interesting that when we knockdown 14-3-3ζ, Sufu mRNA and protein levels are both reduced, indicating that Sufu is a downstream target gene of 14-3-3ζ. Subsequent experiments also demonstrate that Sufu expression is regulated by a 14-3-3ζ binding protein: FoxM1. Both of 14-3-3ζ and FoxM1 regulate Hh signal downstream specific target genes and EMT markers. In our protein turnover assay, we find that FoxM1 stability is required for 14-3-3ζ through ubiquitin-proteasome pathway [33, 34]. The above results indicate that 14-3-3ζ upregulates Sufu transcriptional level is depend on FoxM1 stabilization, so as to active Hh signaling pathway and ultimately accelerate the progression of cervical cancer EMT.

Finally, we analyze the association between overexpression of Sufu and clinical characteristics in cervical cancer tissue microarray to explore the possibility of clinical diagnosis of Sufu. The expression of Sufu is correlated with differentiation grade, FIGO stage, Depth of stromal invasion, vascular cancer embolus. It is possible for Sufu as a new diagnostic marker and therapeutic target for CSCC.

## MATERIALS AND METHODS

### Plasmids, siRNA and CSCC and normal tissues

The Sufu-GFP plasmid was generous gift from Dr. SY Cheng laboratory. The 14-3-3ζ-DDK plasmid was purchased from OriGene. siRNA were all purchased from Invitrogen, siSufu-1#:GGCAGCUUGAGAGCGUACAUCUGAA;siSufu- 2#:GGAUUUAGAAGAUUUGACUUCUCCA;siSufu-3 #: CCCUUGGACUAUGUUAGCAUGUACA.siFoxM1-1#:CCCUGCCCAACAGGAGUCUAAUCAA;siFoxM1-2#:GCCAUGAUACAAUUCGCCAUCAACA;siFoxM1-3#:CGCAUGACUUUGAAAGACAUCUAUA.si14-3-3ζ1#: ACAGCACGCUAAUAAUGCAAUUACU;si14-3-3ζ2#: GAGAGACAACUUGACAUUGUGGACA.Si14-3-3ζ3#: GGAUACCCAAGGAGACG- AAGCUGAA; 88 CSCC and 13 normal cervical tissue specimens used for IHC were obtained from patients without receiving any preoperative chemotherapy or radiotherapy at Jiangxi Provincial Maternal and Child Health Hospital from January 2008 to January 2013. Then multiple tissue microarrays were made from the paraffin-embedded sections.

### Immunohistochemistry and judgment of results

The paraffin-embedded tissue microarrays sections were baked in the oven at 65°C for 12 h. After deparaffinization and blocking, the antigen–antibody reaction was incubated overnight at 4°C. The primary anti-Sufu rabbit monoclonal antibody (Cat No: ab52913, Abcam), anti-14-3-3ζ (Cat No: ab51129, Abcam) and anti-FoxM1(C-20) (Cat No: sc-502, Santa Cruz Biotechnology) were all used at a dilution of 1:100. Two independent pathologists (Y.N.Q and D.M.H) who were blinded to the clinicopathological information and corresponding slides of patients evaluate the immunohistochemical staining of Sufu, 14-3-3ζ and FoxM1. A semiquantitative scoring method was followed: based on the staining intensity (0, negative staining; 1, weak staining; 2, moderate staining; 3, strong staining) and the proportion of immunopositive cells (1, <25%; 2, 25–50%; 3, 50–75%; 4, ≥75%). The final score for Sufu, 14-3-3ζ and FoxM1 expression was the production of the two above-mentioned scores, ranging from 0 to 12. For the statistical analysis, a final staining index of 0–4 represented negative expression, whereas a staining index of 5–12 represented positive expression.

### Protein turnover assay

To measure turnover of endogenous protein, SiHa cell was transfected with control and 14-3-3ζ siRNA, respectively. 48 hours later, the cells were treated with cycloheximide (CHX, 20um, Sigma) to block protein synthesis. At the end of each time point, the cells were lysed in RIPA buffer (50 mM Tris-HCl, pH 7.5, 150mM 475 M NaCl, 1% Nonidet P-40, 0.5% sodium deoxycholate, 0.1% SDS, 1xRoche complete Protease Inhibitor Cocktail))for western analysis.

### Chromatin immunoprecipitation (ChIP) assay

ChIP was carried out as described previously [10]. Chromatin was cross-linked with 1%formaldehyde. Cells were incubated in ChIP lysis buffer (150mM NaCl, 25 mM Tris pH 7.5, 1% Triton X-100, 0.1% SDS, 0.5% deoxycholate, 1xRoche complete Protease Inhibitor Cocktail). The reaction was stopped by the addition of 125 mM glycine. DNA was fragmented into ∼200bp-∼500bp pieces using a Sonics VCX130 sonicator. Aliquots of lysates containing 400 μg of protein were used for each immunoprecipitation reaction with anti-FoxM1 (Cat No: sc-502, Santa Cruz Biotechnology). Precipitated genomic DNA was amplified by PCR with primers as follows. Binding site (BS): Forward 5’-ATGAATCAATCCATTGTCAG-3’; Reverse 5’- ACAGGCAGGGAAGGT- GT-3’.UP_5k: Forward 5’-AGCTGCTCCTATCTTACACTATTC-3’, Reverse 5’- CCCATTAAGTTCACAGCCTTTC-3’.

### Immunoprecipitation

SiHa cells or transfected HEK293T were lysed in IP buffer (50 mM Tris-HCl, pH 7.5, 150mM NaCl, 1%TritonX-100, 1xRoche complete Protease Inhibitor Cocktail), and the lysates were clarified by centrifugation for 15 min at 14,000xg. The protein concentration of each cell lysate sample was determined by BCA assay. Immunoprecipitation was carried out with anti-FoxM1 (Cat No: sc-502, Santa Cruz) or anti-GFP (Cat No: MA5-15256, Thermo Fisher), coupled to Protein G agarose beads (Millipore, USA) and the isolated proteins were subjected to 8% SDS-PAGE, and followed by Western analysis.

### Immunofluorescence

Approximately 1x10^5^ cells were seeded in one well of a Lab-TEK chambered slide. For each siRNA transfected described, 48h later, cells were harvested and washed with PBS, fixed with 4% PFA at 4°C for 10 minutes, then washed with PBS again. Immunofluorescence staining of EMT markers with following antibodies: anti-Vimentin (Cat No: 10366-1-AP, Proteintech) and anti-E-cadherin (Cat No: 610181, BD, Biosciences). Fluorescence was visualized with Olympus X71.

### RNA isolation, RT and real-time PCR

RNA isolation was carried out as described previously [35]. Total RNAs were isolated from cultured cells using the RNAiso reagent (TaKaRa, Shiga, Japan), and reverse transcription reaction was carried out using the PrimeScript RT reagent Kit (TaKaRa).Real-time PCR was carried out using the FastStart SYBR Green Master mix (Roche) on a 7500 Real-Time PCR System (Applied Biosystems) with primers for: RT-hPtch1-F':GCTGCACTACTTCAGAGACTGG, RT-hPtch1-R': CACCAGGAGTTTGTAGGCAAGG. RT- hSufu-F': CAGCAAACCTGTCCTTCCACCA, RT-hSufu-R': CAGATGTACGCTCTCAAGCTGC.RT-hGli1-F': AGCCTT CAGCAATGCCAGTG- AC, RT-hGli1-R':GTCAGGACC ATGCACTGTCTTG.RT-hFoxM1-F': GGAGCAGCGAC AGGT- TAAGG, RT-hFoxM1-R':GTTGATGGCGAA TTGTATCATGG.RT-hActin-F': ACCTTCTACAA- TGAG CTGCG, RT-hActin-R':CCTGGATAGCAACGTACATGG.RT-h14-3-3ζ-F':CTACCGTTA- CTTGGCTGAGG, RT-h14-3-3ζ-R': CCAGTCTGATAGGATGTGTTGG. RT-hE-cadherin-F': CCCAATACATCTCCCTTCACAG, RT-hE-cadherin-R’: CCACCTCTAAGGCCATCTTTG. RT-hVim-F':CGTGAATACCAAGACCTGCTC, RT-hVim-R': GGAAAAGTTTGGAAGAGGCA- G. Experiments were repeated at least three times, and samples were analyzed in triplicates.

### Cell migration and invasion assays

Cell migration and invasion assays were carried out as described previously [36]. For assessing SiHa and HCC94 cell migration, 2x10^4^ cells in serum free media were seeded into the transwell inserts (Corning) and allowed to migrate toward 10% FBS-containing medium. Later, the cells in the transwell inserts were removed, and the inserts were washed in PBS three times. The migrated cells on the bottom of the insert were fixed with methanol solution followed by crystal violet (1%) staining. After washing the inserts three times with PBS, the inserts were allowed to air dry and pictures were taken using Olympus X71. Six independent fields were counted for each transwell and the average number of penetrating cells was represented in the graphs. For assessing cell invasion, 2x10^4^ cells in serum-free medium were seeded in the matrigel-coated transwell inserts (BD Bioscience). The cells were later processed similar to that of cell migration assay.

### Luciferase reporter assay

HEK297T Cells were transfected with the Sufu human promoter reporter plasmid together with pRL-TK and analyzed as described previously [10]. Luciferase activity was measured using a Dual-luciferase assay system (Promega, Madison, WI).

### Statistical analyses

Statistical analyses were performed using SPSS 18.0 software. The results of IHC were analyzed with χ^2^ test and Spearman rank correlation test. All p-values were two-tailed, and p-values less than 0.05 were considered to indicate statistical significance.

## SUPPLEMENTARY MATERIALS FIGURES


