# Supplementary material for: Suppressor of fused (Sufu) promotes epithelial-mesenchymal transition (EMT) in cervical squamous cell carcinoma
Source: Oncotarget. 2017 Dec 11;8(69):114226–38. doi: 10.18632/oncotarget.23176 (PMC5768398; doi:10.18632/oncotarget.23176)
Supplement: Supplementary file 1 [file oncotarget-08-114226-s001.pdf]

Suppressor of fused (Sufu) promotes epithelial-mesenchymal transition (EMT) in cervical squamous cell carcinoma

SUPPLEMENTARY MATERIALS

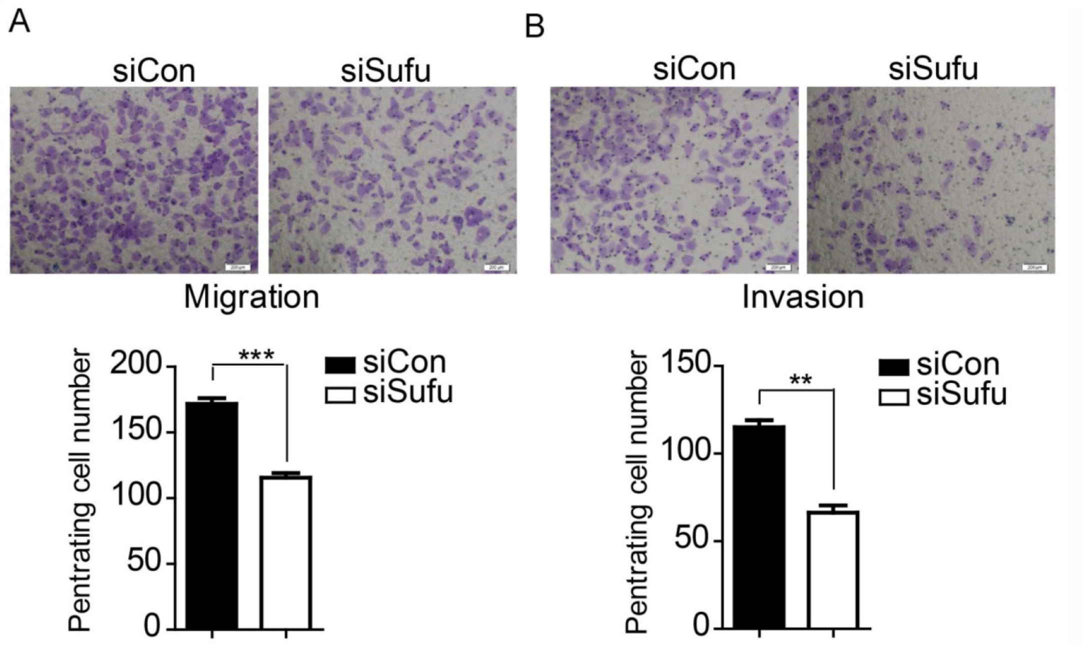

Supplementary Figure 1: Sufu knockdown inhibits HCC94 cells migration and invasion. **(A)** and **(B)** HCC94 cells were transfected with control siRNA or siSufu. After 24 hours of transfection, cells were starved for 24h before cell migration and invasion assays were performed with or without matrigel transwell filters. The invaded or migrated cells were stained and counted. Each bar indicates mean±s.d. of a representative experiment performed in triplicate. P-values were determined by Student's t-test. ** $p<0.01$, *** $p<0.001$.

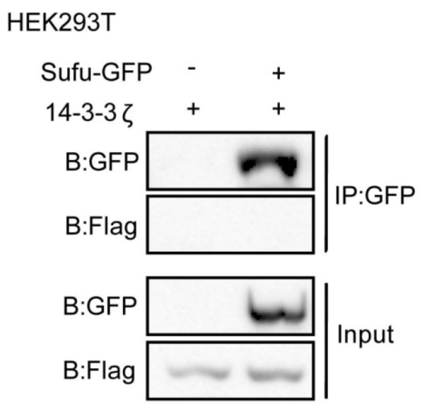

Supplementary Figure 2: Sufu does not bind to 14-3-3ζ. Transfect 14-3-3ζ-Flag construct with Sufu-GFP or control vector into HEK293T, after 24h, cells were harvested and performed Co-IP experiment.

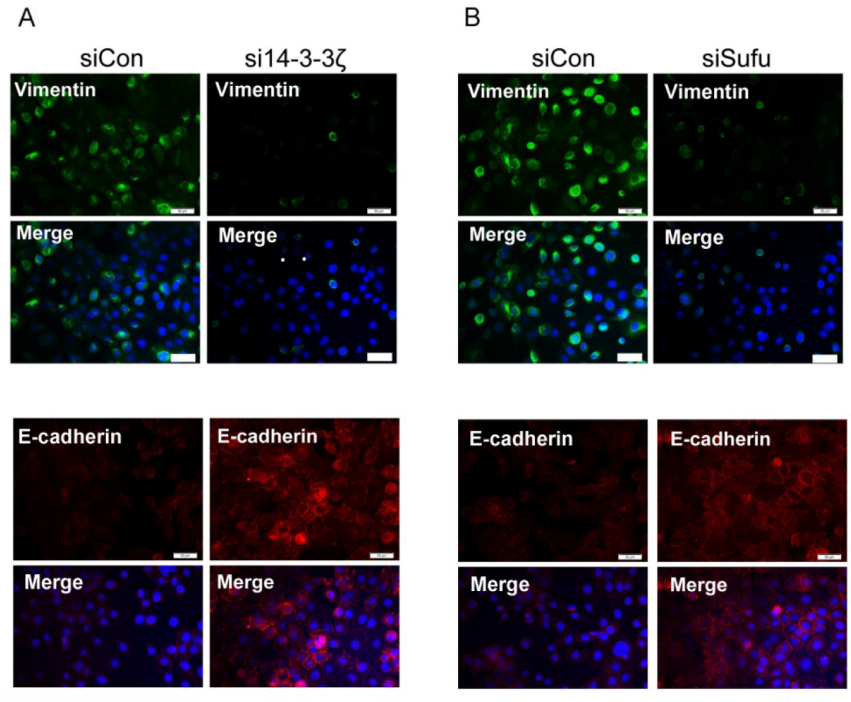

Supplementary Figure 3: Knockdown 14-3-3ζ or Sufu inhibit Vimentin expression but increase E-cadherin expression. HCC94 cells were transfected with control siRNA or 14-3-3ζ **(A)** or siSufu **(B)**. Immunofluorescence staining of epithelial (E-cadherin, Red) and mesenchymal (Vimentin, Green) markers was visualized by microscopy. DAPI staining was included to visualize the cell nucleus (Blue). Scale bar=100 μm.

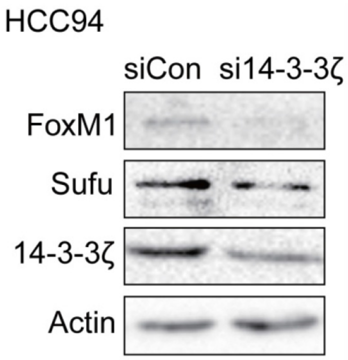

Supplementary Figure 4: Knockown 14-3-3ζ decreases FoxM1 and Sufu expression in HCC94 cell. Western blot analysis showing effects of siFoxM1 on protein levels of Sufu and FoxM1 in HCC94 cell. Actin was used as the loading control.

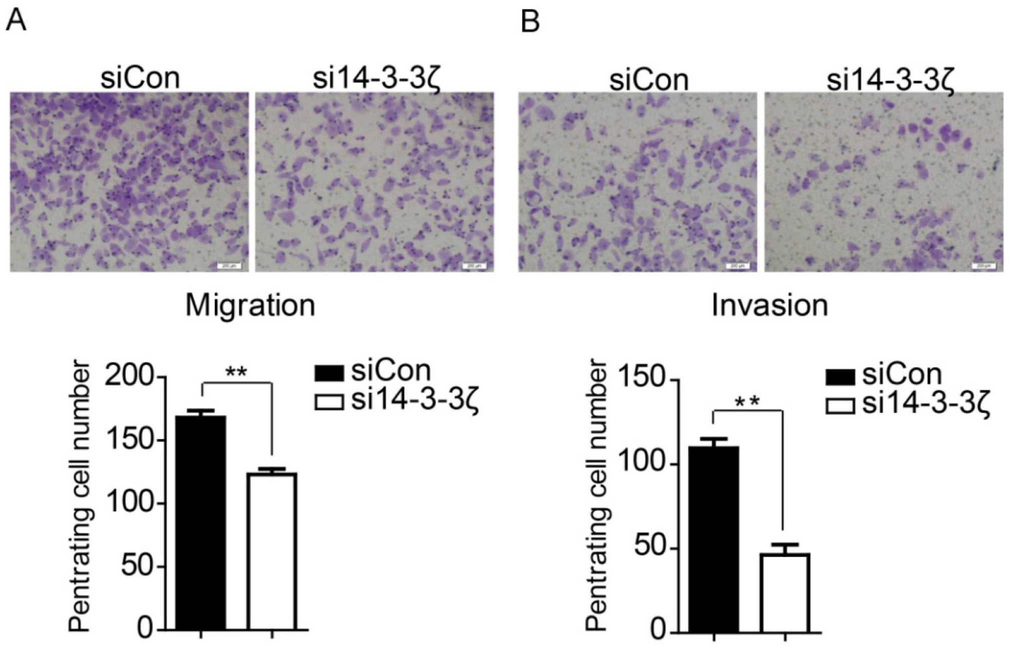

Supplementary Figure 5: Knockdown 14-3-3ζ inhibits HCC94 cells migration and invasion. **(A)** and **(B)** HCC94 cells were transfected with control siRNA or si14-3-3ζ. After 24 hours of transfection, cells were starved for 24h before cell migration and invasion assays were performed with or without matrigel transwell filters. The invaded or migrated cells were stained and counted. Each bar indicates mean±s.d. of a representative experiment performed in triplicate. P-values were determined by Student's t-test. ** $p<0.01$.

A

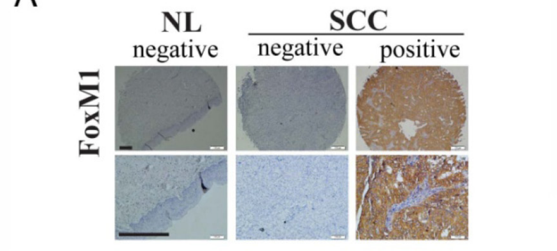

B

Biomarkers	NL n(%)	SCC n(%)	p value
FoxM1	0/13(0)	60/88(68.2)	<0.001

C

		FoxM1 Negative	FoxM1 Positive	Total
14-3-3 ζ	Negative	13	6	19
	Positive	15	54	69
Total		28	60	88

rs=0.412 p<0.001

D

		FoxM1 Negative	FoxM1 Positive	Total
Sufu	Negative	14	8	22
	Positive	14	52	66
Total		28	60	88

rs=0.367 p<0.001

Supplementary Figure 6: FoxM1 and 14-3-3ζ, FoxM1 and Sufu are positively correlated in human tumor tissues. **(A)** A representative image showing the expression of FoxM1 in NL and CSCC tissues. Scale bar=400μm. **(B)** Statistic analysis of immunohistochemistry (IHC) staining of FoxM1 from human CSCC tissue microarray. **(C)** Statistic analysis for FoxM1 and 14-3-3ζ correlation from the IHC staining results in human CSCC tissue microarray. **(D)** Statistic analysis for FoxM1 and Sufu correlation from the IHC staining results in human CSCC tissue microarray.

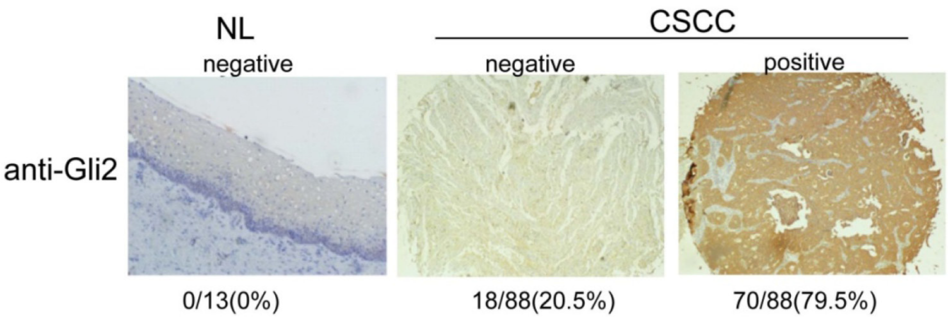

Supplementary Figure 7: Gli2 is overexpressed in CSCC tissue array by IHC. A representative image showing the expression of Gli2 in NL and CSCC tissues.

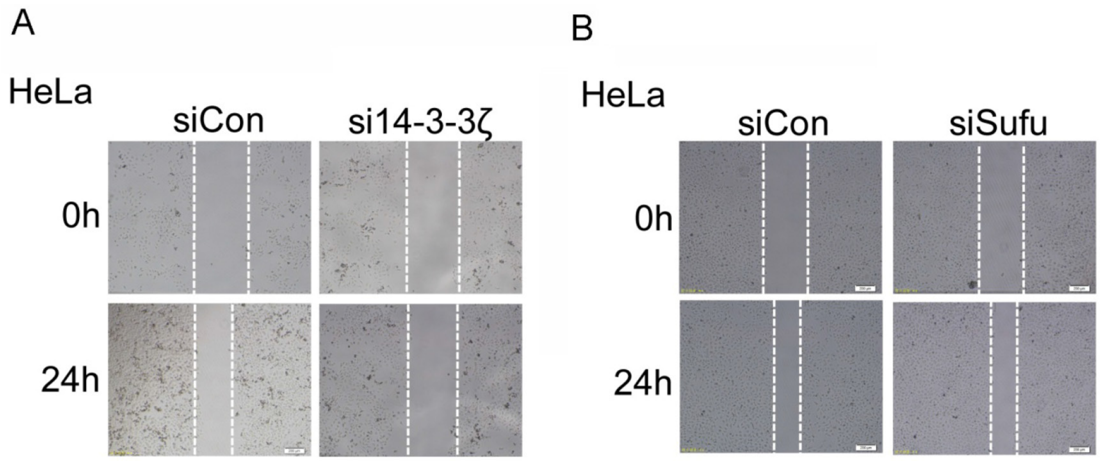

Supplementary Figure 8: Different functions of 14-3-3ζ and Sufu in HeLa cell. **(A)** Wound-healing assay showing that si14-3-3ζ inhibits HeLa cells migration within 24 hours in serum-free medium. **(B)** Wound-healing assay showing that siSufu does not inhibit HeLa cells migration within 24 hours in serum-free medium. Scale bar=200 μm.
